# Recent migrants’ perspectives on antibiotic use and prescribing in primary care: a qualitative study

**DOI:** 10.3399/bjgp16X686809

**Published:** 2016-08-31

**Authors:** Antje Lindenmeyer, Sabi Redwood, Laura Griffith, Shazia Ahmed, Jenny Phillimore

**Affiliations:** Institute for Applied Health Research;; School of Social and Community Medicine, University of Bristol, Bristol.; Health Services Management Centre;; Institute for Applied Health Research;; Institute of Research into Superdiversity, University of Birmingham, Birmingham.

**Keywords:** antibacterial agents, migrants and transients, prescriptions, primary health care, qualitative research

## Abstract

**Background:**

Currently there is great interest in antibiotic prescribing practices in the UK, but little is known about the experiences of the increasing numbers of recent migrants (those present in the UK for >1 year but <5 years) registered at GP practices. Qualitative research has suggested that reasons for not prescribing antibiotics may not be clearly communicated to migrants.

**Aim:**

This study aimed to explore the factors that shape migrants’ experiences of and attitudes to antibiotics, and to suggest ways to improve effective communication around their use.

**Design and setting:**

A qualitative study on recent migrants’ health beliefs, values, and experiences in a community setting in primary care.

**Method:**

Twenty-three recent migrants were interviewed in their preferred language by trained community researchers. The research team conducted a thematic analysis, focusing on health beliefs, engaging with health services, transnational medicine, and concepts of fairness. Experiences around antibiotics were a strong emerging theme.

**Results:**

Three reasons were identified for antibiotics seeking: first, holding an ‘infectious model’ of illness implying that antibiotics are required quickly to avoid illness becoming worse or spreading to others; second, reasoning that other medications will be less effective for people ‘used to’ antibiotics’; and third, perceiving antibiotic prescription as a sign of being taken seriously. Some participants obtained antibiotics from their country of origin or migrant networks in the UK; others changed their mind and accepted alternatives.

**Conclusion:**

Primary care professionals should aim to understand migrants’ perspectives to improve communication with patients. Further research is needed to identify different strategies needed to respond to the varying understandings of antibiotics held by migrants.

## INTRODUCTION

There is a worldwide consensus on the need for restricting antibiotic prescribing to avoid increasing antimicrobial resistance, with initiatives such as Antibiotic Awareness Week promoting best practice.^1^ Recognition of the increasing threat of antimicrobial resistance has given rise to the call for ‘antimicrobial stewardship’, which should include development of resources to facilitate optimal use of antibiotics.^2^ With the most antibiotics for human use prescribed in primary care, GPs are important in promoting their judicious use.^3^ Good doctor–patient communication is crucial as antibiotics may be prescribed inappropriately if the doctor feels unable, or lacks time, to explain why antibiotics are not a suitable treatment.^4^ Non-prescribing of antibiotics needs to be managed carefully as a prescription is *‘a significant and valued event’* signalling the end of the consultation and its successful conclusion,^5^ and nonprescription may lead to reduced patient satisfaction.^6^ A Europe-wide consortium; Genomics to Combat Resistance against Antibiotics in Community-acquired lower respiratory tract infections (LRTIs) in Europe (GRACE) has developed an Internet-based training tool for LRTIs that was found to be acceptable and applicable by physicians in several European countries.^7^ In the UK, recent NICE guidance recommends that prescribers should elicit patients’ concerns and whether they want or expect an antimicrobial.^8^ Interventions such as the TARGET toolkit^9^ or the interactive booklet ‘When should I worry’^10^ have been used successfully in the consultation to aid discussion and make the best use of consultation time.^5^

However, none of these interventions are aimed at recent migrants. Translated versions, for example, of the TARGET leaflet, are available in languages spoken by large migrant groups only (for example, Arabic, Polish, and Urdu). The advent of migration-driven superdiversity,^11^ however, means that more migrants are arriving from more places, to more areas in the UK, than ever before; the 2011 census showed that the number of people born overseas and living in England and Wales had risen to one person in eight.^12^ Inevitably, more recently-arrived migrants will register with primary care services in the NHS, some encountering a primary care service for the first time in their lives.^13^^,^^14^ Although medication taking is always embedded in a cultural context,^15^ availability of antibiotics is much greater in most countries outside Europe and North America, where between 19% and 100% of antibiotics are taken without a prescription.^3^ Within Europe, antibiotics are available without prescription from some pharmacies, for example in Spain,^16^ Greece,^17^ and Poland,^18^ where higher availability may also drive higher prescribing.^7^ Many recently-arrived migrants also come from conflict zones or areas with poor water quality, sanitation, and nutrition, where a lack or delay in access to antibiotics for infectious diseases such as pneumonia may kill more people than antibiotic resistance.^19^ Therefore, recent migrants’ experiences of antibiotic taking can be very different both from white British populations and established minority ethnic groups.

**How this fits in**

Good patient–GP communication is essential to promote judicious use of antibiotics. However, qualitative research has found that communicating prescribing decisions with migrant patients may be more difficult. In this study, recent migrants discussed their experiences of taking antibiotics that were very different from those of white British patients or established minorities. Primary care professionals should aim to understand migrants’ perspectives to improve communication and identify different strategies required for appropriate response.

In UK general practice, prescription rates for antibiotics, after levelling off around the year 2000, have increased again in recent years.^20^ One might speculate whether the arrival of migrants who have previously been able to access antibiotics, or who are seeking access having previously experienced a lack, could be a factor driving this rise. Indeed, this would make a good topic for future research.

Routinely-collected practice data in primary care is unlikely to establish whether this is the case; although practices are meant to capture when a new patient joins the NHS from overseas, country of origin data are limited and currently not routinely analysed by clinical commissioning groups,^21^ recording of ethnic group is also imperfect,^22^ and research on prescription patterns using proxy data has been inconclusive.^23^^,^^24^ Therefore, it is important to explore recent migrants’ own perspectives on antibiotic use to understand who seeks antibiotics and why. Although there is comparatively little research on recent migrants’ experiences of health care in the UK, a 2008 study on asylum seekers found that not being able to get antibiotics was a major source of dissatisfaction;^25^ participants in a thesis study on African migrants said that they had tried another GP or the walk-in centre when refused antibiotics, while some had obtained antibiotics from families abroad.^26^ This study aimed to explore the factors shaping migrants’ experiences of and attitudes to antibiotics, and to suggest ways to improve communication around their use.

## METHOD

This research forms part of a qualitative study on recent migrants’ health beliefs, values, and experiences of health care. The focus was on recent migrants as previous work has demonstrated that newness is a key factor in shaping the ways that migrants engage with health providers.^13^ The study aim was to understand the migrants’ recent experience of moving to a different health system and its implications for healthcare interactions. Interviews were conducted with 23 migrants who had been in the UK for >1 year, but <5 years, aiming for maximum variation according to region of origin, immigration status, age, and sex (Table 1).

**Table 1.** Demographics of participants

**Sex**	**Country of birth**	**Age, years**	**Immigration status**	**Reason for move**	**Language of interview**	**Antibiotics discussed**
Female	Iran	30	Leave to remain	Seeking asylum	Farsi	Yes
Female	Iran	40	Unclear	Joining spouse	Farsi	No
Female	Iran	35	Refugee	Seeking asylum	Farsi	No
Male	Iran	39	Refugee	Seeking asylum	Farsi	Yes
Female	Poland	25	EU migrant	Study	Polish	No
Male	Poland	36	EU migrant	Work	Polish	Yes
Female	Poland	36	EU migrant	Work	Polish	Yes
Female	Poland	52	EU migrant	Joining daughters	Polish	Yes
Female	Pakistan	26	British citizenship	Marriage	Urdu	No
Female	Pakistan	28	British citizenship	Marriage	Urdu	No
Male	India	34	British citizenship	Marriage	Gujarati	Yes
Female	India	34	British citizenship	Marriage	Gujarati	Yes
Male	Iraq	37	Leave to remain	Seeking asylum	English	No
Male	Somalia	29	Leave to remain	Family reunion	Somali (interpreted)	Yes
Female	Zimbabwe	60	Leave to remain	Seeking asylum	Shona	Yes
Female	Sudan	29	Asylum seeker	Seeking asylum	Zaghawa (interpreted)	Yes
Male	Cameroon	26	Asylum seeker	Seeking asylum	French	No
Male	Cameroon	30	Unclear	Unclear	French	No
Female	DR Congo	30	Asylum seeker	Seeking asylum	French	No
Female	Uganda	26	Leave to remain	Seeking asylum	Luganda	No
Male	Ivory Coast	35	Asylum seeker	Seeking asylum	French	Yes
Female	China	38	Leave to remain	Joining spouse	Cantonese	No
Female	China	25	Leave to remain	Joining spouse	Mandarin	Yes

This approach was designed to elicit a range of perspectives but also identify commonalities between very different groups. Interviews were conducted in the participants’ preferred language by trained community researchers with the relevant language and cultural competence. Community researchers also transcribed and translated the interviews and contributed to the analysis. The research team collectively conducted a thematic analysis of the transcripts,^27^ focusing on health beliefs, interaction with health services, fairness and justice, and the use of health resources from abroad and the UK. It was found that interviewees often raised the use of antibiotics as an issue when asked about their healthcare experience and a number of themes developed related to the role of antibiotics in shaping encounters with primary care services in the UK.

## RESULTS

Two researchers led on the analysis of responses mentioning antibiotics, with two others checking the analysis against the extracted quotes. Out of 23 interviewees, 12 specifically discussed their use of antibiotics in their country of origin and/or antibiotic prescribing in the UK, while most other participants talked about asking for prescription only medications (POMs).

Antibiotics were described as widely available over the counter or on prescription in participants’ countries of origin. Generally, there was a sense of a ‘health marketplace’ where consumers could access most medications if they could afford to pay. Some participants from African countries also indicated that people on a low income in their country had to resort to unregulated medication sellers:

‘Here, everything is organised … in my country people are corrupt and will cheat you with fake medicine.’

(Male, 29, from Somalia)

The main themes arising from all the interview accounts were:

- the curative power of antibiotics and other ‘strong’ medicines;
- a prescription of antibiotics indicating being taken seriously by the GP; and
- alternatives to antibiotics.

### The curative powers of antibiotics and ‘strong’ medicines

Participants who discussed their experiences with antibiotics in their country of origin often stressed their efficacy in helping them to *‘get better quickly’* (Female, aged 34, from India), especially when given as an injection. One participant assumed that the power of antibiotics could deal with all her health problems:

‘ … in Iran when we became ill, with one visit to the doctor and with the first injection they gave us they would revive us.’

(Female, 30, from Iran)

At the same time, participants stressed that antibiotics had to be given quickly before the infection could get worse or spread to others:

*‘I had frostbites which became septic but I couldn’t have antibiotics because in this country, it takes a long time until you are given antibiotics, yet back home in our countries, as long as there is sepsis or infection, even high temperature antibiotics are given. And with us when we come from abroad we would be used to antibiotics.* […] *I couldn’t* [retrain as a nurse] *because my fingers were septic and I was afraid again to as I have to be aware of infecting other people.’*

(Female, 60, from Zimbabwe)

This speedy approach to treatment was often contrasted with descriptions of the slow pace of the health system in the UK. Not being given POMs immediately was a source of dissatisfaction even for participants who were largely positive about the health service in the UK, implying that a ‘watch and wait’ approach before prescribing medications may be a difficult concept for some.

Several participants were concerned that they were used to ‘strong’ medications and that ‘weak’ medications such as paracetamol would not work for them. There was some blurring between antibiotics and analgesics in the descriptions of strong medicine but some very clearly believed that they needed antibiotics:

‘ … our bodies need strong medication … the medication here is very weak’.

(Female, 30, from Iran)

*‘It’s better for* [doctors] *to see if people are from abroad and if they had been used to antibiotics.’*

(Female, 60, from Zimbabwe)

‘In Africa, when they take out your teeth, they give you medication … like antibiotic, pain killer!’

(Male, 35, from Ivory Coast)

The desire for antibiotics should be seen in the context of many migrants being in a more general state of disease because of the cold weather, the strain of moving to an unknown country, and being uncertain whether they would be able to remain:

*‘Since I arrived here, I have had constant headache, unable to do any exercise, and also as a result of the cold wintry weather, I would remain indoors for hours on end … Any time I receive a letter from the Home Office or my solicitor* [about her asylum case], *I always feel sick and worried.’*

(Female, 29, from Sudan)

Another participant felt that migrating to the UK had left her in a generally weakened state in which strong medicine would be needed, with antibiotic injections specifically mentioned as having the power to cure:

Participant (P): ‘*When we returned from Turkey* […] *I was ill for 5 months.*

Interviewer (I): Did you go to your GP?

P: I want, I said give us an injection. They said we don’t do that here.

I: What injection?

P: An injection like in Iran, Penicillin for example

I: Ah, antibiotics.

P: *Yes, with that my problem would have been solved. Since I came to England I am constantly ill because my body has become weak, because they are giving me weak medicines.* (Female, 30, from Iran)

### Being taken seriously

This theme deals with the perception that healthcare professionals in the UK often disregarded, or misunderstood, migrants’ health concerns in a way that made the health service resemble a ‘paracetamol service’.^28^ One reason for this view may be that a prescription was the expected outcome of a consultation:

*‘What more could I expect than* [doctors] *giving me a check-up and then prescribing medication for me to take?’*

(Female, 34, from India)

Being ‘sent away with a paracetamol’ instead of an antibiotic or other POMs signified to some of the participants that their problem was not being taken seriously by the GP:

‘When you go to the surgery, the doctor will see you only for one illness and only for a short time. If you tell them that you have a headache, they will ask you to buy paracetamol over the counter.’

(Female, 29, from Sudan)

‘You really have to inflate how you are for them to care. If you just go and say I have a pain here, from start to finish you get a paracetamol. They won’t do anything else for you.’

(Female, 40, from Iran)

Participants’ misgivings could be compounded when they did not understand *why* they were being refused antibiotics:

‘I can’t just buy antibiotics, and many times when I have gone to the GP and he just tells me, well, you have a cold and that medication everyone jokes about (laughs) … You say my head, leg, stomach or anything is hurting, they give you paracetamol.’

(Male, 39, from Iran)

*‘If I ask* [doctors] *to prescribe antibiotics for example when I have a cough, or chest pain, they will not give it to you, instead they will say it is not good for your health.’*

(Female, 29, from Sudan)

These responses show a significant breakdown in communication. Although we could not access GPs’ notes or recollections, these participants’ stories indicate that any attempts to explain why antibiotics could not be prescribed were unsuccessful, and the participants left the consultation disappointed and somewhat confused.

### Alternatives to antibiotic prescriptions

Participants’ decisions about how they would go about accessing health services were informed by their earlier experiences with primary care.

An unsatisfactory experience could lead to seeking help elsewhere. A few dismissed primary care services out of hand:

‘The GPs are not helpful … you don’t exist for them.’

(Female, 35, from Iran)

‘I have no trust in the doctors here.’

(Female, 40, from Iran)

*‘We* [Africans] *need to constantly ask the GPs to give us stronger medications.’*

(Male, 26, from Cameroon)

Some participants looked for alternatives or chose to seek assistance from friends and relatives in their home countries, or migrant networks in the UK:

‘I’ve never been really ill but all my mates go to a private Polish doctor, because doctors here don’t prescribe medication, just paracetamol for everything.’

(Male, 36, from Poland)

*I constantly get medication from my family in Iran, such as amoxicillin.* […] *Here I don’t buy medication. Even a syrup for a cold I ask them to bring it for my child from Iran. I don’t believe in their medicines here.’*

(Female, 30, from Iran)

These responses are a cause for concern as patients may take antibiotics, possibly in conjunction with other UK-prescribed medications, without informing their GP:

*They don’t do anything here, just prescribe paracetamol for everything.* […] *The only thing I went to a doctor for here was a chest infection once, but the doctor asked me to wait until it will go away by itself! Can you believe it? The doctor said 2 weeks of heavy cough is not enough to prescribe medication. Luckily I can get all needed medication in the Polish shop; you can even find antibiotics there. Otherwise I don’t know what I would do.’*

(Female, 52, from Poland)

There was evidence, however, to suggest that some participants had reconsidered their views on antibiotics:

*‘* [In the UK] *the doctor doesn’t easily give you antibiotics. I will declare in all honesty that back home the moment I felt I had a cold I would go to the pharmacy and buy amoxicillin and take it. Well this is totally wrong because your body becomes resistant to antibiotics and then when you really need antibiotics then it won’t work.’*

(Male, 39, from Iran)

‘I know now that if you’re prescribed antibiotics straight away then your body can become immune to them.’

(Male, 34, from India)

*‘When we were in China,* [the doctor] *gave medicines immediately, although it gets better quicker, the side effect is still quite heavy. Normally in the UK,* [doctors] *will not just give you any medicines for kids. Even if going to pharmacies to buy medicines like antibiotics, they would not give me those.* […] *If it were not a serious illness, the GP would not give a prescription.’*

(Female, 25, from China)

Interestingly, the first two participants had picked up a misconception also widely held in Western countries, which is that people who use antibiotics themselves could become ‘immune’ to them.^29^ Although it is not entirely clear what caused the change in opinion, words like ‘side effects’ or ‘resistance’ might indicate that these participants looked at patient leaflets or talked to their GP.

### Different perspectives on antibiotics

While participants’ accounts reflected their own experiences, three main approaches to antibiotics were outlined linked to the healthcare system and health issues in their country of origin:

- the ‘infectious model’ with a focus on the need to take antibiotics quickly to cure illness and prevent further infection (represented in this sample by participants from African countries and India);
- the ‘strong medicine’ model with a focus on the powerful effect on the individual, which could include strong side effects (participants from Iran and China and some African countries); and
- seeking antibiotics when illness persists (participants from Poland).

## DISCUSSION

### Summary

The present study offers some insights into the expectations of new migrants regarding access to antibiotics, the reasons why some may seek antibiotics, and why discussions around them can be so fraught. First, some had personal experience of the curative power of antibiotics and the necessity of antibiotics to be given quickly to avoid deterioration and further spread of infection. Second, there was a more general expectation that a consultation should conclude with a prescription and that this is a sign of good care and the patient’s illness being taken seriously. Additionally, frustration with the GP service meant that some patients obtained antibiotics from their country of origin or migrant networks in the UK. Others changed their mind, however, and accepted self-care advice or alternatives to antibiotics.

From the migrants’ point of view, the desire for antibiotics is entirely reasonable given many of their earlier experiences. Persuading them that an antibiotic is not needed may be challenging. Given the diversity of migrant populations, primary care professionals need to understand the diversity of health experiences, beliefs, and associated expectations, and the potential consequences of not addressing these.

Alongside existing guidelines stressing the importance of discussing patients’ worries and concerns, awareness of this diversity could contribute to improved communication and increased trust in primary care services.

### Strengths and limitations

To the authors’ knowledge, this article is the first to focus on recent migrants’ perspectives on antibiotics in the UK. it will become increasingly important to understand their experiences as the patient population becomes more diverse and the need to find effective ways of discussing antibiotics in the consultation becomes more acute. Although antibiotics emerged as a theme, the study findings should be viewed as tentative given that community researchers did not probe for further reflection when participants raised the issue. Clinical reasons for delaying the prescription of antibiotics were often described as an example of delays in the system with more general experiences of delays in the system, and therefore were hard to disaggregate. The study sample was unable to represent ‘all recent migrants’; a group that will change constantly because of the nature of migration flows in an era of superdiversity. It was possible, however, to discern approaches to antibiotics that were consistent in wider groups of participants. The small sample size and emergent nature of the antibiotics theme meant that it was not possible to achieve saturation for these categories; further research is needed for confirmation or further development.

### Comparison with existing literature

The present research illustrates the challenges of providing appropriate services for highly diverse populations, which have been widely acknowledged.^30^^,^^31^ As recently as 2008, a general British population had similar high expectations, using terms such as ‘quick’, ‘effective’, ‘strong’, and ‘life savers’ when describing antibiotics.^32^ For many recent migrants, the experience of having been able to easily access antibiotics in their home countries appeared to be the main difference. The ‘infectious model’ of illness held by many African migrants to the UK^26^ could be relevant to patients from other regions, however, where life-threatening bacterial infections are endemic and where there is a lack of needed antibiotics. The present study suggested that this model may lead to a higher demand for antibiotics as patients believe that they need to be taken early in illness to prevent deterioration and infecting others.

While there are clear health implications for patients if they take antibiotics without a prescription,^33^ the consequences of unsatisfactory consultations related to the use of antibiotics have a more general potential for disrupting doctor–patient relationships, especially where migrants do not understand clearly why they have been refused antibiotics and interpret such refusal as a lack of concern for their problems. In their study of asylum seekers’ experiences of health care in Scotland, O’Donnell and colleagues describe how concerns about antibiotics arose in addition to more general dissatisfaction with waiting times and short consultations; they also outline how feeling that pain or persisting respiratory illness is not taken seriously can increase the likelihood of losing faith in primary care.^34^ Additionally, there are wider social expectations common to many health systems where the prescription of POMs is taken to represent a successful end to the consultation or to legitimise the presenting illness as warranting medical attention.^35^

A general sense of dissatisfaction may proliferate further when migrants share their experiences via their networks. A study from Holland describes how the term ‘paracetamol’ is used in Somali communities as shorthand for having one’s health problems dismissed by the doctor.^36^ The present study participants also indicated that discussions about the problems they experienced when seeking antibiotics may be commonplace in migrant communities (for example, everybody ‘joking’ about paracetamol). This kind of talk, although accurately reflecting their experiences, has the potential to prevent new migrants in their networks from seeking medical attention from GPs if they feel that their perceived needs would not be taken seriously.^28^

### Implications for research and practice

Given the current concerns around antimicrobial resistance coupled with the increase in migrants who may have very different experiences, action is needed to improve the quality of communication around antibiotic prescribing. The challenge will be to seek to change understanding and expectations without new migrants feeling they have to resort to alternative sources of medication. The actions recommended for a general patient population (eliciting patient worries, taking time to explain a decision) are especially important here. Additionally, GPs could use different strategies depending on the general approach to antibiotics; for example, reassuring patients with an ‘infectious model’ that their condition will not rapidly become more serious if they are refused antibiotics and to advise that in the UK viruses are generally manageable without medication, or exploring with the patient whether ‘strong medicine’ is really needed now. Given that some participants accepted that antibiotics could potentially be harmful to them and to the wider community, there is evidence that it is possible to reassure patients. Stressing traditional ways of avoiding infection (for example, getting enough rest, taking vitamins, and eating nutrient-rich foods such as chicken soup) as well as building up resilience may be more successful than attempts to limit antibiotic use directly.^37^ For example, one of our participants recalled that the doctor advised him to ‘*let the body fight the infection’* rather than taking medication.

Participants in the present study employed similar common-sense models when asked what promotes good health (such as, a good diet, adequate fresh air and exercise, a harmonious family life, and an absence of stress and worry), which offers potential avenues for further exploration. The development of educational toolkits that take into account different experiences of antibiotic use could aid communication around antibiotic use as well as equip future generations of doctors to work with increasingly diverse patient populations.
